# Supplementary material for: Discovery and application of insertion-deletion (INDEL) polymorphisms for QTL mapping of early life-history traits in Atlantic salmon
Source: BMC Genomics. 2010 Mar 8;11:156. doi: 10.1186/1471-2164-11-156 (PMC2838853; doi:10.1186/1471-2164-11-156)
Supplement: Additional file 2 — Information on developed 76 locus single-run INDEL panel in Atlantic salmon. Information on fluorescence labeling, primer concentrations, PCR pooling and links to alignments, INDEL motifs and GENESCAN (Burge and Karlin 1997) predictions of genes/exons are available in html format. [file 1471-2164-11-156-S2.ZIP › Additionalfile2/table21745.html]

Contig Alignment

```
Base	A B C D   
1	G . G .    
2	C . C .    
3	C . C .    
4	A . A .    
5	T . T .    
6	A . A .    
7	T . T .    
8	T . T .    
9	C . C .    
10	A . A .    
11	T . T .    
12	G . G .    
13	C . C .    
14	A . A .    
15	G . G .    
16	A . A .    
17	A . A .    
18	A . A .    
19	C . C .    
20	A . A .    
21	C . C C    
22	A . A A    
23	A . A A    
24	A . A A    
25	A . A A    
26	C . C C    
27	A . A A    
28	T . T T    
29	G . G G    
30	A . A A    
31	T . T T    
32	T . T T    
33	T . T T    
34	A . A A    
35	G . G G    
36	A . A A    
37	T . T T    
38	T . T T    
39	T . T T    
40	G . G G    
41	T . T T    
42	T . T T    
43	T . T T    
44	A . A A    
45	T . T T    
46	G . G G    
47	A . A A    
48	T . T T    
49	G . G G    
50	T . T T    
51	A . A A    
52	A . A A    
53	A . A A    
54	G . G G    
55	A . A A    
56	A . A A    
57	T . T T    
58	T . T T    
59	G . G G    
60	A . A A    
61	A . A A    
62	T . T T    
63	A . A A    
64	A . A A    
65	A . A A    
66	C . C C    
67	C . C C    
68	A . A A    
69	C . C C    
70	C . C C    
71	T . T T    
72	T . T T    
73	G . G G    
74	G . G G    
75	G . G G    
76	G . G G    
77	G . G G    
78	G . G G    
79	G . G G    
80	T . T T    
81	T . T T    
82	A . A A    
83	T . T T    
84	G . G G    
85	A . A A    
86	C . C C    
87	C . C C    
88	A . A A    
89	A . A A    
90	A . A A    
91	A . A A    
92	A . A A    
93	T . T T    
94	A . A A    
95	T . T T    
96	G . G G    
97	C . C C    
98	C . C C    
99	C . C C    
100	C . C C    
101	T . T T    
102	A . A A    
103	G . G G    
104	G . G G    
105	G . G G    
106	T . T T    
107	C . C C    
108	A . A A    
109	G . G G    
110	A . A A    
111	T . T T    
112	G . G G    
113	A . A A    
114	C . C C    
115	C . C C    
116	T . T T    
117	T . T T    
118	A . A A    
119	T . T T    
120	G . G G    
121	A . A A    
122	C . C C    
123	C . C C    
124	C . C C    
125	C . C C    
126	A . A A    
127	G . G G    
128	T . T T    
129	C . C C    
130	A . A A    
131	G . G G    
132	A . A A    
133	A . A A    
134	T . T T    
135	C . C C    
136	G . G G    
137	G . G G    
138	A . A A    
139	G . G G    
140	T . T T    
141	T . T T    
142	C . C C    
143	C . C C    
144	A . A A    
145	T . T T    
146	C . C C    
147	T . T T    
148	C . C C    
149	T . T T    
150	C . C C    
151	T . T T    
152	C . C C    
153	T . T T    
154	G . G G    
155	G . G G    
156	A . A A    
157	C . C C    
158	C . C C    
159	C . C C    
160	C . C C    
161	T . T T    
162	T . T T    
163	T . T T    
164	C . C C    
165	C . C C    
166	C . C C    
167	A . A A    
168	G . G G    
169	A . A A    
170	A . A A    
171	C . C C    
172	T . T T    
173	G . G G    
174	T . T T    
175	G . G G    
176	T . T T    
177	G . G G    
178	A . A A    
179	C . C C    
180	T . T T    
181	G . G G    
182	A . A A    
183	C . C C    
184	T . T T    
185	G . G G    
186	A . A A    
187	C . C C    
188	T . T T    
189	C . C C    
190	A . A A    
191	G . G G    
192	C . C C    
193	T . T T    
194	G . G G    
195	C . C C    
196	T . T T    
197	A . A A    
198	G . G G    
199	C . C C    
200	T . T T    
201	A . A A    
202	G . G G    
203	G . G G    
204	C . C C    
205	A . A A    
206	T . T T    
207	C . C C    
208	G . G G    
209	T . T T    
210	G . G G    
211	T . T T    
212	T . T T    
213	C . C C    
214	G . G G    
215	T . T T    
216	T . T T    
217	A . A A    
218	G . G G    
219	C . C C    
220	T . T T    
221	A . A A    
222	T . T T    
223	A . A A    
224	G . G G    
225	C . C C    
226	T . T T    
227	G . G G    
228	T . T T    
229	G . G G    
230	T . T T    
231	A . A A    
232	G . G G    
233	C . C C    
234	G . G G    
235	A . A A    
236	C . C C    
237	T . T T    
238	T . T T    
239	A . A A    
240	G . G G    
241	C . C C    
242	C . C C    
243	A . A A    
244	A . A A    
245	C . C C    
246	T . T T    
247	A . A A    
248	G . G G    
249	G . G G    
250	C . C C    
251	A . A A    
252	A . A A    
253	C . C C    
254	T . T T    
255	C . C C    
256	T . T T    
257	G . G G    
258	C . C C    
259	T . T T    
260	C . C C    
261	T . T T    
262	A . A A    
263	T . T T    
264	G . G G    
265	T . T T    
266	T . T T    
267	C . C C    
268	A . A A    
269	T . T T    
270	A . A A    
271	A . A A    
272	C . C C    
273	T . T T    
274	A . A A    
275	A . A A    
276	A . A A    
277	A . A A    
278	C . C C    
279	T . G G  1	 transversion  
280	A . A A    
281	A . A A    
282	T . T T    
283	A . A A    
284	G . G G    
285	C . C C    
286	T . T T    
287	A A A A    
288	T T T T    
289	T T T T    
290	T T T T    
291	G G G G    
292	C C C C    
293	T T T T    
294	A A A A    
295	T T T T    
296	G G G G    
297	T T T T    
298	T T T T    
299	G G G G    
300	C C C C    
301	G G G G    
302	A A A A    
303	T T T T    
304	G G G G    
305	A A A A    
306	C C C C    
307	A A A A    
308	A A A A    
309	T T T T    
310	A A A A    
311	C C C C    
312	A A A A    
313	T T T T    
314	T T T T    
315	C C C C    
316	A A A A    
317	C C C C    
318	C C C C    
319	T T T T    
320	G G G G    
321	G G G G    
322	A A A A    
323	T T T T    
324	A A A A    
325	T T T T    
326	T T T T    
327	A A A A    
328	G G G G    
329	C C C C    
330	T T T T    
331	A A A A    
332	A A A A    
333	T T T T    
334	T T T T    
335	G G G G    
336	C C C C    
337	C C C C    
338	A A A A    
339	G G G G    
340	G G G G    
341	G G G G    
342	T T T T    
343	A A A A    
344	G G G G    
345	G G G G    
346	T T T T    
347	A A A A    
348	G G G G    
349	C C C C    
350	T T T T    
351	A A A A    
352	A A A A    
353	T T T T    
354	G G G G    
355	A A A A    
356	A A A A    
357	C C C C    
358	T T T T    
359	C C C C    
360	C C C C    
361	C C C C    
362	C C C C    
363	T T T T    
364	G G G G    
365	G G G G    
366	A A A A    
367	G G G G    
368	T T T T    
369	A A A A    
370	T T T T    
371	A A A A    
372	A A A A    
373	A A A A    
374	C C C C    
375	T T T T    
376	A A A A    
377	G G G G    
378	A A A A    
379	A A A A    
380	C C C C    
381	A A A A    
382	T T T T    
383	C C C C    
384	T T T T    
385	A A A A    
386	C C C C    
387	G G G G    
388	A A A A    
389	T T T T    
390	C C C C    
391	A A A A    
392	T T T T    
393	G G G G    
394	A A A A    
395	A A A A    
396	G G G G    
397	C C C C    
398	A A A A    
399	G G G G    
400	A A A A    
401	G G G G    
402	C C C C    
403	C C C C    
404	A A A A    
405	G G G G    
406	A A A A    
407	C C C C    
408	A A A A    
409	G G G G    
410	A A A A    
411	A A A A    
412	T T T T    
413	T T T T    
414	C C C C    
415	C C C C    
416	T T T T    
417	G G G G    
418	C C C C    
419	C C C C    
420	T T T T    
421	A A A A    
422	G G G G    
423	G G G G    
424	G G G G    
425	A A A A    
426	T T T T    
427	G G G G    
428	A A A A    
429	G G G G    
430	A A A A    
431	G G G G    
432	C C C C    
433	G G G G    
434	C C C C    
435	A A A A    
436	T T T T    
437	C C C C    
438	A A A A    
439	T T T T    
440	T T T T    
441	G G G G    
442	C C C C    
443	A A A A    
444	C C C C    
445	C C C C    
446	G G G G    
447	G G G G    
448	T T T T    
449	T T T T    
450	A A A A    
451	A A A A    
452	C C C C    
453	A A A A    
454	C C C C    
455	C C C C    
456	G G G G    
457	A A A A    
458	A A A A    
459	G G G G    
460	C C C C    
461	A A A A    
462	T T T T    
463	G G G G    
464	C C C C    
465	C C C C    
466	T T T T    
467	T T T T    
468	T T T T    
469	C C C C    
470	C C C C    
471	T T T T    
472	C C C C    
473	A A A A    
474	C C C C    
475	A A A A    
476	T T T T    
477	G G G G    
478	C C C C    
479	A A A A    
480	C C C C    
481	A A A A    
482	A A A A    
483	T T T T    
484	T T T T    
485	G G G G    
486	T T T T    
487	A A A A    
488	A A A A    
489	A A A A    
490	C C C C    
491	A A A A    
492	A A A A    
493	C C C C    
494	A A A A    
495	T T T T    
496	A A A A    
497	A A A A    
498	A A A A    
499	C C C C    
500	A A A A    
501	C C C C    
502	A A A A    
503	T T T T    
504	C C C C    
505	C C C C    
506	A A A A    
507	G G G G    
508	G G G G    
509	C C C C    
510	G G G G    
511	C C C C    
512	T T T T    
513	T T T T    
514	G G G G    
515	G G G G    
516	C C C C    
517	T T T T    
518	G G G G    
519	C C C C    
520	A A A A    
521	C C C C    
522	C C C C    
523	A A A A    
524	T T T T    
525	G G G G    
526	T T T T    
527	T T T T    
528	T T T T    
529	T T T T    
530	A A A A    
531	C C C C    
532	T T T T    
533	G G G G    
534	G G G G    
535	A A A A    
536	C C C C    
537	G G G G    
538	T T T T    
539	A A A A    
540	T T T T    
541	A A A A    
542	T T T T    
543	G G G G    
544	G G G G    
545	C C C C    
546	C C C C    
547	C C C C    
548	T T T T    
549	A A A A    
550	A A A A    
551	C C C C    
552	T T T T    
553	T T T T    
554	C C C C    
555	A A A A    
556	T T T T    
557	G G G G    
558	G G G G    
559	A A A A    
560	C C C C    
561	G G G G    
562	A A A A    
563	G G G G    
564	G G G G    
565	G G G G    
566	A A A A    
567	T T T T    
568	A A A A    
569	A A A A    
570	A A A A    
571	A A A A    
572	C C C C    
573	A A A A    
574	C C C C    
575	T T T T    
576	G G G G    
577	G G G G    
578	G G G G    
579	T T T T    
580	C C C C    
581	A A A A    
582	C C C C    
583	A A A A    
584	G G G G    
585	G G G G    
586	G G G G    
587	A A A A    
588	T T T T    
589	G G G G    
590	A A A A    
591	G G G G    
592	A A A A    
593	G G G G    
594	C C C C    
595	- - C C  2	 indel  
596	- - G G  2	 indel  
597	- - C C  2	 indel  
598	G G G G    
599	C C C C    
600	A A A A    
601	A A A A    
602	A A A A    
603	A A A A    
604	T T T T    
605	G G G G    
606	G G G G    
607	A A A A    
608	C C C C    
609	A A A A    
610	A A A A    
611	C C C C    
612	T T T T    
613	G G G G    
614	T T T T    
615	T T T T    
616	T T T T    
617	A A A A    
618	T T T T    
619	G G G G    
620	T T T T    
621	T T T T    
622	G G G G    
623	T T T T    
624	C C C C    
625	C C C C    
626	A A A A    
627	A A A A    
628	G G G G    
629	T T T T    
630	G G G G    
631	G G G G    
632	C C C C    
633	A A A A    
634	T T T T    
635	G G G G    
636	A A A A    
637	A A A A    
638	T T T T    
639	C C C C    
640	A A A A    
641	G G G G    
642	A A A A    
643	G G G G    
644	T T T T    
645	T T T T    
646	T T T T    
647	G G G G    
648	C C C C    
649	A A A A    
650	C C C C    
651	A A A A    
652	A A A A    
653	A A A A    
654	A A A A    
655	T T T T    
656	G G G G    
657	T T T T    
658	A A A A    
659	T T T T    
660	T T T T    
661	T T T T    
662	T T T T    
663	G G G G    
664	T T T T    
665	T T T T    
666	C C C C    
667	G G G G    
668	C C C C    
669	A A A A    
670	T T T T    
671	T C C C  1	 transition  
672	. G G G    
673	. T T T    
674	. A A A    
675	. A A A    
676	. A A A    
677	. C C C    
678	. A A A    
679	. T T T    
680	. G G G    
681	. C C C    
682	. A A A    
683	. T T T    
684	. C C C    
685	. A A A    
686	. T T T    
687	. T T T    
688	. C C C    
689	. A A A    
690	. G G G    
691	. T T T    
692	. T T T    
693	. T T T    
694	. T T T    
695	. G G G    
696	. A A A    
697	. C C C    
698	. T T T    
699	. G G G    
700	. C C C    
701	. T T T    
702	. A A A    
703	. G G G    
704	. T T T    
705	. G G G    
706	. A A A    
707	. G G G    
708	. G G G    
709	. C C C    
710	. T T T    
711	. C C C    
712	. C C C    
713	. T T T    
714	. G G G    
715	. G G G    
716	. C C C    
717	. T T T    
718	. T T T    
719	. T T T    
720	. T T T    
721	. C C C    
722	. A A A    
723	. G G G    
724	. G G G    
725	. A A A    
726	. T T T    
727	. A A A    
728	. T T T    
729	. T T T    
730	. G G G    
731	. A A A    
732	. A A A    
733	. A A A    
734	. T T T    
735	. T T T    
736	. A A A    
737	. C C C    
738	. T T T    
739	. T T T    
740	. A A A    
741	. G G G    
742	. C C C    
743	. C C C    
744	. T T T    
745	. A A A    
746	. A A A    
747	. A A A    
748	. T T T    
749	. T T T    
750	. A A A    
751	. G G G    
752	. G G G    
753	. G G G    
754	. G G G    
755	. T T T    
756	. T T T    
757	. T T T    
758	. T A A  1	 transversion  
759	. . G G    
760	. . C C    
761	. . A A    
762	. . C C    
763	. . G G    
764	. . C C    
765	. . C C    
766	. . C C    
767	. . G G    
768	. . C C    
769	. . T T    
770	. . C C    
771	. . C C    
772	. . C C    
773	. . T T    
774	. . A A    
775	. . C C    
776	. . A A    
777	. . A A    
778	. . A A    
779	. . C C    
780	. . C C    
781	. . A A    
782	. . C C    
783	. . C C    
784	. . C C    
785	. . . C    
786	. . . C
```
